# Current Views Regarding the Tonsils and Their Surgical Removal

**Published:** 1917-04

**Authors:** 


### CURRENT VIEWS REGARDING THE TONSILS AND THEIR SURGICAL REMOVAL

---

Nowadays when any organ of the body attains a previously unsuspected importance so that considerable professional attention is concentrated on it, the circumstances is soon reflected in the interest and expectations of the layman. This is seen, for example, in the popular attitude toward the tonsils and adenoids. Few untutored persons have any conception as to the purpose, not to say the location and anatomic relations, of these lymphoid tissues. The physican may counsel the removal of one of the structures with a clear understanding that he is urging what is, in a sense, an experimental operation which may remove a possible cause of disease in other organs. The layman, encouraged by the apparent innocuousness of the operative procedure, soon begins to look on tonsillectomy as a sort of panacea for the most diverse ills; and somehow this state of mind occasionally seems to weigh in the balance with the professional man himself. That the uses of these lymphoid structures are so little understood is no reasôn for counting every prominent tonsil or adenoid in persons of school age as a defect. Indeed, there seems to be a wholesome reaction against the operations on tonsils in youth merely because they are prominent and noticeable. At the Johns Hopkins Hospital, for example, the practice is to regard the tonsils and adenoids in children as physiologically important parts of the mechanism which protects the lower air passages from dust and organisms.[1] If there are no mouth-breathing, no evidence of damage to the ears, no chronic enlargement of the glands of the neck, no cystic condition of the adenoids known as Thornwald's disease, and none of the so-called "reflex neuroses," the removal of adenoids regardless of their size or appearance is not recommended.

On the other hand, recent years have witnessed a growing interest in management of diseased tonsils and a widespread appreciation that the germ of tonsilitis may hit in many spots far removed from the local seat of obvious disorder. Tonsillar and nasopharyngeal infections have of late frequently become related, for example, to infec-

---

[1] Crowe, S. J.; Watkins, S. S., and Rothholz, Alma S.: Relation of Tonsillar and Nasopharyngeal Infections to General Systemic Disorders, Bull. Johns Hopkins Hosp., 1917, 28, 1.

tious and rheumatoid arthritis, myositis and acute rheumatic fever, to chorea, to hyperplasia of the cervical glands, and even to nephritis. If these relationships and other indications of the involvement of the tonsils as portals of entry for harmful microorganisms could be convincingly established, the indications for the removal of the tonsils both as a prophylactic and as a therapeutic measure would become clearer and more rational. It is of unusual value, therefore, to have available a carefully digested study of the relation of tonsillar and nasopharyngeal infections to general systemic disorders, based on 1,000 cases in which operation was performed at the Johns Hopkins Hospital during the past five years.[1] An added importance is lent by the fact that these operations were not done in a haphazard way in hurried dispensary service without experienced anesthetists or assistants; they were performed under the rigorous routine of a surgical ward, and the cases were followed up in order to learn the subsequent history with respect to disorders supposed to be secondary to a chronic focus of infection in the upper air passages.

As a result of the experience now collated, Dr. Crowe and his associates have reached certain tentative conclusions which may be helpful in the contemplation of tonsillectomy. They assert that the operation should never be undertaken during the acute stage of tonsillar inflammation, as a cerebral abscess may result. Diabetes is as much a contraindication for tonsillectomy as it may be for any operation necessitating general anesthesia. Tonsillectomy is rarely of benefit in the chronic deforming types of arthritis, in many cases probably doing more harm than good. The Baltimore surgeons are further convinced that nothing is to be gained from a tonsillectomy during the acute stage of chorea, acute rheumatic fever or endo-

---

1 Crowe, S. J.; Watkins, S. S., and Rothholz, Alma S.: Relation of Tonsillar and Nasopharyngeal Infections to General Systemic Disorders, Bull. Johns Hopkins Hosp., 1917, 28, 1.

carditis. Their experience shows that even after the nose and throat have been put in normal condition by operative measures these diseases may recur. In any event the tonsils are not the only portals of entry for the etiologic organism, and their removal in an interval free from symptoms can be justified only on the plea of preventing further cardiac lesions which may result from subsequent acute tonsilitis. The frequency of heart and joint defects in chorea may justify such a prophylactic measure.

In infectious arthritis in which the periarticular changes predominate, and perhaps also in myalgia, the promise of helpfulness from tonsillectomy is greater. In the early stages of glomerulonephritis it may also be worthy of some consideration. The tonsils are the most common site of the chronic infections which give rise to a hyperplasia of the deep cervical lymph glands near the angle of the jaw. The enlargement of the glands will rarely subside after treatment of carious teeth and alveolar abscesses alone. It seems advisable, therefore, under such conditions to consider the removal of the tonsils in cases of persistent palpable glands at the angle of the jaw, particularly if the patient has some general systemic disorder. The majority of the enlargements are apparently due to a chronic pyogenic infection which will subside after tonsillectomy. The others are due, as a rule, to persistent tubercle bacilli.

Obviously the advisability of a tonsillectomy in any individual case depends on the malady and the general condition of the patient. As the Baltimore investigators express it, tonsillectomy alone will not cure tuberculous cervical adenitis, arthritis or glomerular nephritis. It is necessary in such cases to apply general hygienic measures as well, so as to increase the patient's resistance. If the tonsils are the primary focus of infection, their removal in suitable instances may materially alter the prognosis by preventing a constant reinfection. Skillful surgery is indispensable. A partial occlusion of the crypts, resulting from an incomplete tonsillectomy, as sometimes happens, may actually aggravate the symptoms of infection by producing a mechanically made focus of trouble. Despite the many uncertainties and unsolved problems which still exist, however, Crowe and his collaborators state that their records "tend to support the evidence of Billings and others in regard to the importance of focal infections in many of the general disorders seen by the internist, the pediatrician and the general surgeon."—Editorial, *Journal A. M. A.*, March 17, 1917.

